# Book Review: Studies on Cognitive Processes in Translation

**DOI:** 10.3389/fpsyg.2021.736197

**Published:** 2021-08-18

**Authors:** Qiangfu Yu

**Affiliations:** Faculty of Humanities and Foreign Languages, Xi'an University of Technology, Xi'an, China

**Keywords:** cognitive processes, translation, psycholinguistics, cognitive translatology, cognitive science

Since Krings (1986) first proposed that the focus of translation studies is to understand what happens in the translator's mind, studies on cognitive processes in translation have been carried out for more than three decades. Holmes (1988/2000) predicted that high precision methods developed by psychologists would promote the development of translation process research, a branch of descriptive translation studies. During the recent 10 years, the field of translation process research has made considerable development, and gradually formed two kinds of cognitive research paradigms: cognitive research paradigm of psychology and cognitive linguistics. *Studies on Cognitive Processes in Translation* aims to integrate these two research paradigms and systematically explore the theories, methods and frontier issues of studies on cognitive processes in translation.

The book has five chapters. Chapter 1 reviews the development course, main objectives and research contents of studies on cognitive processes in translation both in China and abroad, and points out that there are two generations of language cognitive views in the research of cognitive translatology, namely, the first generation of classic objectivism cognition and the second generation of connectionism and situated/embedded/embodied cognition. The author proposes that from the perspective of the second generation of cognitive science, the primary goal of studies on cognitive processes in translation is to focus on more cognitive psychological processes involved in translation, expand and deepen the understanding of the translator's internal cognitive behaviors, and explore deeper secrets in the brain.

Chapter 2 mainly discusses the research methods applied in studies on cognitive processes in translation. According to the data sources and measurement indicators, the author classifies research methods of cognitive processes in translation into five categories: verbal reports, behavioral measurement, physiological measurement, text analysis, and supplementary methods. This chapter introduces the application of the above five categories in the studies on cognitive processes in translation, and analyzes the advantages and disadvantages of each research method. Finally, it discusses the feasibility and prospect of multi-element mutual verification/triangulating in the studies on translation processes.

Chapter 3 focuses on the descriptive and explanatory models of cognitive processes in translation. In view of lack of consistency in the theoretical framework of the existing studies on cognitive processes in translation, this chapter, based on the macro perspective of cognitive science, makes a clearer positioning of the theoretical starting points, perspectives and presuppositions reflected in various cognitive process models. It focuses on the influential translation process models, such as Bell's translation process model, Alves and Gonçalves' relevance-connectionism model, PACTE translation competence model, translatorial cognition and action network, Kintsch's construction-integration model, hence making clear the future research directions.

Chapter 4 reviews the existing researches on translator's real-time cognitive processing with several core constructs as the main line, and comments on the basic assumptions, cognitive dimensions, and limitations. Based on the second generation of cognitive science paradigm, especially from the perspective of cognitive linguistics, this chapter also looks forward to the future of real-time cognitive processing of translation.

Chapter 5 first explores the influences of the changes of translation workspace and the development of translation technology on the translation processes, and then discusses the theoretical frameworks of contextualized cognition, socio-cultural cognition, affective cognition, unconscious cognition, and common cognitive process in translation, as well as the existing empirical researches, and looks into the trends of future researches.

Chapter 6 is the conclusion of the whole book, which summarizes the relevant research fields and problems mentioned in the above chapters, and discusses how to meet the challenges and promote the studies on cognitive processes in translation to the unknown field. The author argues that cognitive translatology must innovate and integrate its methodology; the importance of conceptual research and theoretical research should not be ignored; it is necessary to explore the practical influences or applications of studies on cognitive processes in translation and interpretation.

As a monograph exploring the studies on cognitive processes in translation, this book embodies the trend of interdisciplinary research, that is, the research methods and new technologies of psycholinguistics and cognitive science are constantly applied to the studies on cognitive processes in translation, realizing the mutual benefit and win-win development among disciplines. As far as the research content is concerned, this book has the characteristics of both theoretical reviews and experimental researches. The theoretical review not only reviews and rethinks the related researches in this field, but also points out the problems to be solved or the research trend, which has important reference value for future researches. As for experimental researches, this book expands the scope of studies on cognitive processes in translation and deepens the research of old topics. It is one of the representative works reflecting the current research progress of studies on cognitive processes in translation. This book is characterized by distinct theme, rigorous structure, and strong objectivity. Therefore, it is these qualities that make *Studies on Cognitive Processes in Translation* an excellent book with both practicability and collectability for post-graduate students, researchers, and scholars interested in studies on cognitive processes in translation.

However, there still remain some respects worth Tan Yesheng's further elaboration. Firstly, although the experimental studies in this book mention the application of neuroimaging techniques, such as electroencephalogram, functional magnetic resonance imaging, and current skin test, in the study of cognitive processes in translation, these currently frequently-applied methods in the fields of psycholinguistics and cognitive science are not expounded in details. Secondly, the book does not involve the reception study in the processes of translation. In other words, it only deals with the processes of the translator's understanding, transformation and expression, rather than the reception process of the target readers. Finally, the book would be more comprehensive if it included more studies in China.

## Author Contributions

QY has written the book review and approved the submitted version for publication.

## Conflict of Interest

The author declares that the research was conducted in the absence of any commercial or financial relationships that could be construed as a potential conflict of interest.

## Publisher's Note

All claims expressed in this article are solely those of the authors and do not necessarily represent those of their affiliated organizations, or those of the publisher, the editors and the reviewers. Any product that may be evaluated in this article, or claim that may be made by its manufacturer, is not guaranteed or endorsed by the publisher.
